# Supplementary material for: Multi‐dataset identification of innovative feature genes and molecular mechanisms in keratoconus
Source: J Cell Mol Med. 2024 Sep 19;28(18):e70079. doi: 10.1111/jcmm.70079 (PMC11412914; doi:10.1111/jcmm.70079)
Supplement: Supplementary file 2 — Material S2. [file JCMM-28-e70079-s002.zip › 7.GSEA analysis/2.ATOH7/KEGG_WNT_SIGNALING_PATHWAY.html]

Details for gene set KEGG\_WNT\_SIGNALING\_PATHWAY[GSEA]

|  || Dataset | ATOH7.group.cls#h\_versus\_l |
| Phenotype | group.cls#h\_versus\_l |
| Upregulated in class | h |
| GeneSet | KEGG\_WNT\_SIGNALING\_PATHWAY |
| Enrichment Score (ES) | 0.37296435 |
| Normalized Enrichment Score (NES) | 1.5710323 |
| Nominal p-value | 0.007677543 |
| FDR q-value | 0.28817457 |
| FWER p-Value | 0.67 |
Table: GSEA Results Summary

  

Fig 1: Enrichment plot: KEGG\_WNT\_SIGNALING\_PATHWAY      
 Profile of the Running ES Score & Positions of GeneSet Members on the Rank Ordered List

  

| SYMBOL | TITLE | RANK IN GENE LIST | RANK METRIC SCORE | RUNNING ES | CORE ENRICHMENT || 1 | WNT1 | na | 172 | 0.697 | 0.0274 | Yes |
| 2 | PRICKLE2 | na | 246 | 0.648 | 0.0554 | Yes |
| 3 | AXIN2 | na | 285 | 0.630 | 0.0836 | Yes |
| 4 | MMP7 | na | 328 | 0.614 | 0.1109 | Yes |
| 5 | WNT9A | na | 551 | 0.534 | 0.1293 | Yes |
| 6 | LEF1 | na | 735 | 0.492 | 0.1469 | Yes |
| 7 | WNT6 | na | 825 | 0.474 | 0.1663 | Yes |
| 8 | PRKACB | na | 829 | 0.474 | 0.1882 | Yes |
| 9 | NKD1 | na | 862 | 0.470 | 0.2091 | Yes |
| 10 | WNT5B | na | 1090 | 0.433 | 0.2227 | Yes |
| 11 | NFATC1 | na | 1442 | 0.392 | 0.2308 | Yes |
| 12 | SOX17 | na | 1648 | 0.373 | 0.2422 | Yes |
| 13 | PLCB1 | na | 1718 | 0.367 | 0.2573 | Yes |
| 14 | PRKACG | na | 1784 | 0.361 | 0.2722 | Yes |
| 15 | PRKCB | na | 2281 | 0.324 | 0.2729 | Yes |
| 16 | CCND2 | na | 2406 | 0.315 | 0.2839 | Yes |
| 17 | CAMK2D | na | 2442 | 0.313 | 0.2975 | Yes |
| 18 | FZD7 | na | 2482 | 0.310 | 0.3108 | Yes |
| 19 | WNT9B | na | 2657 | 0.300 | 0.3197 | Yes |
| 20 | WNT11 | na | 3016 | 0.280 | 0.3224 | Yes |
| 21 | TCF7 | na | 3059 | 0.277 | 0.3340 | Yes |
| 22 | FZD10 | na | 3060 | 0.277 | 0.3469 | Yes |
| 23 | NFATC4 | na | 3293 | 0.266 | 0.3525 | Yes |
| 24 | WNT7A | na | 3390 | 0.261 | 0.3619 | Yes |
| 25 | WNT8B | na | 3424 | 0.259 | 0.3730 | Yes |
| 26 | FZD2 | na | 4571 | 0.216 | 0.3499 | No |
| 27 | VANGL2 | na | 4953 | 0.203 | 0.3483 | No |
| 28 | APC | na | 5634 | 0.182 | 0.3370 | No |
| 29 | WNT7B | na | 5905 | 0.174 | 0.3373 | No |
| 30 | WNT10A | na | 6547 | 0.156 | 0.3260 | No |
| 31 | TCF7L1 | na | 6644 | 0.153 | 0.3303 | No |
| 32 | PPP3CC | na | 7238 | 0.137 | 0.3196 | No |
| 33 | WNT3A | na | 7555 | 0.129 | 0.3164 | No |
| 34 | PRKCA | na | 7938 | 0.120 | 0.3110 | No |
| 35 | FZD4 | na | 8076 | 0.117 | 0.3125 | No |
| 36 | WNT2 | na | 8209 | 0.114 | 0.3140 | No |
| 37 | DKK1 | na | 8242 | 0.114 | 0.3183 | No |
| 38 | PPP2CB | na | 8608 | 0.106 | 0.3127 | No |
| 39 | NFAT5 | na | 8760 | 0.103 | 0.3131 | No |
| 40 | DVL2 | na | 9335 | 0.093 | 0.3008 | No |
| 41 | PPP2CA | na | 9540 | 0.090 | 0.2991 | No |
| 42 | EP300 | na | 10138 | 0.079 | 0.2855 | No |
| 43 | CACYBP | na | 10313 | 0.076 | 0.2840 | No |
| 44 | TBL1X | na | 10621 | 0.072 | 0.2785 | No |
| 45 | CCND3 | na | 10699 | 0.071 | 0.2795 | No |
| 46 | FOSL1 | na | 10739 | 0.070 | 0.2817 | No |
| 47 | APC2 | na | 10749 | 0.070 | 0.2847 | No |
| 48 | WNT4 | na | 10868 | 0.068 | 0.2844 | No |
| 49 | PPP2R5B | na | 11069 | 0.065 | 0.2816 | No |
| 50 | PPP3CA | na | 11626 | 0.057 | 0.2682 | No |
| 51 | PPP3CB | na | 11848 | 0.055 | 0.2644 | No |
| 52 | FZD8 | na | 11987 | 0.053 | 0.2628 | No |
| 53 | MAP3K7 | na | 12201 | 0.050 | 0.2590 | No |
| 54 | PPP2R1A | na | 12268 | 0.049 | 0.2594 | No |
| 55 | PRKACA | na | 12347 | 0.048 | 0.2593 | No |
| 56 | CSNK1A1L | na | 12390 | 0.048 | 0.2603 | No |
| 57 | SFRP5 | na | 12417 | 0.047 | 0.2618 | No |
| 58 | SIAH1 | na | 12493 | 0.046 | 0.2618 | No |
| 59 | PRKX | na | 12697 | 0.044 | 0.2579 | No |
| 60 | PPP2R5C | na | 12732 | 0.043 | 0.2589 | No |
| 61 | DVL3 | na | 12971 | 0.041 | 0.2540 | No |
| 62 | TBL1Y | na | 13081 | 0.040 | 0.2526 | No |
| 63 | CSNK2B | na | 13092 | 0.039 | 0.2542 | No |
| 64 | CSNK2A2 | na | 13289 | 0.037 | 0.2503 | No |
| 65 | DVL1 | na | 13325 | 0.037 | 0.2510 | No |
| 66 | FRAT1 | na | 13440 | 0.036 | 0.2493 | No |
| 67 | SENP2 | na | 13493 | 0.035 | 0.2495 | No |
| 68 | RAC2 | na | 13916 | 0.031 | 0.2387 | No |
| 69 | CHP1 | na | 14674 | 0.024 | 0.2180 | No |
| 70 | TP53 | na | 14698 | 0.024 | 0.2184 | No |
| 71 | SKP1 | na | 14712 | 0.024 | 0.2191 | No |
| 72 | WNT8A | na | 15047 | 0.021 | 0.2104 | No |
| 73 | RHOA | na | 15382 | 0.018 | 0.2016 | No |
| 74 | PPP3R1 | na | 15411 | 0.018 | 0.2016 | No |
| 75 | PPP2R5E | na | 15532 | 0.017 | 0.1989 | No |
| 76 | AXIN1 | na | 15654 | 0.015 | 0.1961 | No |
| 77 | GSK3B | na | 16028 | 0.013 | 0.1859 | No |
| 78 | RAC1 | na | 16331 | 0.010 | 0.1776 | No |
| 79 | JUN | na | 16587 | 0.008 | 0.1706 | No |
| 80 | FZD6 | na | 16592 | 0.008 | 0.1709 | No |
| 81 | PPARD | na | 17078 | 0.004 | 0.1571 | No |
| 82 | CUL1 | na | 17124 | 0.003 | 0.1559 | No |
| 83 | MAPK8 | na | 17140 | 0.003 | 0.1556 | No |
| 84 | BTRC | na | 17413 | 0.001 | 0.1478 | No |
| 85 | PORCN | na | 17431 | 0.001 | 0.1473 | No |
| 86 | PPP2R5D | na | 17451 | 0.001 | 0.1468 | No |
| 87 | DAAM1 | na | 17828 | -0.003 | 0.1360 | No |
| 88 | SMAD3 | na | 18063 | -0.005 | 0.1295 | No |
| 89 | CTNNB1 | na | 18214 | -0.006 | 0.1254 | No |
| 90 | CTNNBIP1 | na | 18711 | -0.010 | 0.1116 | No |
| 91 | CXXC4 | na | 19209 | -0.015 | 0.0979 | No |
| 92 | TBL1XR1 | na | 19269 | -0.015 | 0.0969 | No |
| 93 | NLK | na | 19287 | -0.015 | 0.0971 | No |
| 94 | SFRP4 | na | 19359 | -0.016 | 0.0958 | No |
| 95 | ROCK2 | na | 19403 | -0.016 | 0.0953 | No |
| 96 | MYC | na | 19651 | -0.019 | 0.0890 | No |
| 97 | FBXW11 | na | 19927 | -0.021 | 0.0820 | No |
| 98 | FZD9 | na | 19998 | -0.022 | 0.0810 | No |
| 99 | WNT3 | na | 20041 | -0.022 | 0.0808 | No |
| 100 | FRAT2 | na | 20211 | -0.024 | 0.0771 | No |
| 101 | FZD3 | na | 20400 | -0.026 | 0.0728 | No |
| 102 | CCND1 | na | 20546 | -0.027 | 0.0699 | No |
| 103 | PLCB4 | na | 20753 | -0.029 | 0.0653 | No |
| 104 | LRP5 | na | 20896 | -0.031 | 0.0626 | No |
| 105 | MAPK10 | na | 20989 | -0.032 | 0.0614 | No |
| 106 | RUVBL1 | na | 21063 | -0.032 | 0.0608 | No |
| 107 | DKK2 | na | 21095 | -0.033 | 0.0614 | No |
| 108 | FZD5 | na | 21276 | -0.035 | 0.0578 | No |
| 109 | LRP6 | na | 21337 | -0.035 | 0.0577 | No |
| 110 | CHD8 | na | 21548 | -0.038 | 0.0534 | No |
| 111 | RBX1 | na | 21760 | -0.040 | 0.0492 | No |
| 112 | DAAM2 | na | 21993 | -0.043 | 0.0445 | No |
| 113 | SMAD4 | na | 22118 | -0.044 | 0.0429 | No |
| 114 | TCF7L2 | na | 22134 | -0.044 | 0.0446 | No |
| 115 | CAMK2A | na | 22272 | -0.046 | 0.0427 | No |
| 116 | FZD1 | na | 22280 | -0.046 | 0.0447 | No |
| 117 | CTBP2 | na | 22714 | -0.051 | 0.0345 | No |
| 118 | PSEN1 | na | 22990 | -0.055 | 0.0291 | No |
| 119 | NFATC3 | na | 23138 | -0.057 | 0.0275 | No |
| 120 | CAMK2G | na | 23214 | -0.058 | 0.0280 | No |
| 121 | VANGL1 | na | 23389 | -0.060 | 0.0258 | No |
| 122 | PPP2R5A | na | 23614 | -0.064 | 0.0223 | No |
| 123 | PLCB3 | na | 24121 | -0.071 | 0.0109 | No |
| 124 | CTBP1 | na | 24172 | -0.072 | 0.0128 | No |
| 125 | CSNK2A1 | na | 24568 | -0.078 | 0.0050 | No |
| 126 | WNT5A | na | 24650 | -0.080 | 0.0064 | No |
| 127 | MAPK9 | na | 24826 | -0.082 | 0.0052 | No |
| 128 | CREBBP | na | 24831 | -0.083 | 0.0089 | No |
| 129 | SMAD2 | na | 25145 | -0.088 | 0.0039 | No |
| 130 | ROCK1 | na | 25698 | -0.098 | -0.0075 | No |
| 131 | DKK4 | na | 26454 | -0.114 | -0.0240 | No |
| 132 | CSNK1A1 | na | 26580 | -0.117 | -0.0222 | No |
| 133 | WNT16 | na | 27162 | -0.130 | -0.0330 | No |
| 134 | WNT10B | na | 27303 | -0.133 | -0.0309 | No |
| 135 | SFRP2 | na | 27462 | -0.137 | -0.0290 | No |
| 136 | PLCB2 | na | 28336 | -0.160 | -0.0468 | No |
| 137 | CHP2 | na | 28497 | -0.164 | -0.0438 | No |
| 138 | PPP2R1B | na | 28524 | -0.165 | -0.0369 | No |
| 139 | PRICKLE1 | na | 28915 | -0.176 | -0.0400 | No |
| 140 | PRKCG | na | 28978 | -0.178 | -0.0335 | No |
| 141 | NFATC2 | na | 29713 | -0.201 | -0.0454 | No |
| 142 | WNT2B | na | 30926 | -0.244 | -0.0691 | No |
| 143 | NKD2 | na | 31161 | -0.253 | -0.0641 | No |
| 144 | RAC3 | na | 32406 | -0.320 | -0.0852 | No |
| 145 | PPP3R2 | na | 32636 | -0.337 | -0.0762 | No |
| 146 | CER1 | na | 33436 | -0.408 | -0.0803 | No |
| 147 | WIF1 | na | 34179 | -0.536 | -0.0769 | No |
| 148 | CAMK2B | na | 34693 | -0.989 | -0.0457 | No |
| 149 | SFRP1 | na | 34698 | -1.005 | 0.0009 | No |
Table: GSEA details [plain text format]

  

Fig 2: KEGG\_WNT\_SIGNALING\_PATHWAY      
 Blue-Pink O' Gram in the Space of the Analyzed GeneSet

  

Fig 3: KEGG\_WNT\_SIGNALING\_PATHWAY: Random ES distribution      
 Gene set null distribution of ES for **KEGG\_WNT\_SIGNALING\_PATHWAY**
